# Healthcare teams as complex adaptive systems: understanding team behaviour through team members’ perception of interpersonal interaction

**DOI:** 10.1186/s12913-018-3392-3

**Published:** 2018-07-20

**Authors:** Peter Pype, Fien Mertens, Fleur Helewaut, Demi Krystallidou

**Affiliations:** 1 Department of Family Medicine and Primary Health Care, University Hospital – 6K3, Corneel Heymanslaan 10, B-9000 Ghent, Belgium; 2 End-of-Life Care Research Group, Ghent University & Vrije Universiteit Brussel (VUB), Brussels, Belgium; 3 Clinical Skills Training Centre, Faculty of Medicine and Health Sciences, University Hospital 2K3, Corneel Heymanslaan 10, B-9000 Ghent, Belgium; 4 Faculty of Arts (Sint Andries Campus), University of Leuven, Sint Andriesstraat 2, B-2000 Antwerp, Belgium

**Keywords:** Complexity science, Complex adaptive systems, Interprofessional relations, Healthcare teams, Interpersonal interaction, Palliative care, Interdisciplinary communication

## Abstract

**Background:**

Complexity science has been introduced in healthcare as a theoretical framework to better understand complex situations. Interdisciplinary healthcare teams can be viewed as Complex Adaptive Systems (CAS) by focusing more on the team members’ interaction with each other than on the characteristics of individual team members. Viewing teams in this way can provide us with insights into the origins of team behaviour. The aim of this study is to describe the functioning of a healthcare team as it originates from the members’ interactions using the CAS principles as a framework and to explore factors influencing workplace learning as emergent behaviour.

**Methods:**

An interview study was done with 21 palliative home-care nurses, 20 community nurses and 18 general practitioners in Flanders, Belgium. A two-step analysis consisted of a deductive approach, which uses the CAS principles as coding framework for interview transcripts, followed by an inductive approach, which identifies patterns in the codes for each CAS principle.

**Results:**

All CAS principles were identified in the interview transcripts of the three groups. The most prevalent principles in our study were principles with a structuring effect on team functioning: team members act autonomously guided by internalized basic rules; attractors shape the team functioning; a team has a history and is sensitive to initial conditions; and a team is an open system, interacting with its environment. The other principles, focusing on the result of the structuring principles, were present in the data, albeit to a lesser extent: team members’ interactions are non-linear; interactions between team members can produce unpredictable behaviour; and interactions between team members can generate new behaviour. Patterns, reflecting team behaviour, were recognized in the coding of each CAS principle. Patterns of team behaviour, identified in this way, were linked to interprofessional competencies of the Interprofessional Collaboration Collaborative. Factors influencing workplace learning were identified.

**Conclusions:**

This study provides us with insights into the origin of team functioning by explaining how patterns of interactions between team members define team behaviour. Viewing healthcare teams as Complex Adaptive Systems may offer explanations of different aspects of team behaviour with implications for education, practice and research.

## Background

Complexity science belongs to the latest generation systems thinking, studying complex systems [1], also called Complex Adaptive Systems (CAS), by focusing on the relations and interconnections of the system components, rather than on the individual components themselves. Applied to the world of termites, for instance, the communication and collaboration between termites is more important than the characteristics of every individual termite. Complexity science has been introduced in healthcare as a theoretical framework to better understand complex situations [2–5]. Using complexity science to study healthcare has provided insights that could not have been reached when only using the traditional explanatory model in medicine based on scientific positivism that describes the linear cause-effect relationship between two isolated events [1]. The way clinicians handle uncertainty during the diagnostic process, the way physiological processes regulate, for instance, blood glucose levels and the way healthcare practices organize themselves according to a number of simple rules are examples of complex system behaviour that cannot be fully understood through linear thinking alone [1, 3]. As such, many healthcare concepts (e.g. diseases) and systems (e.g. hospitals) have subsequently been described as CAS [1, 6, 7]. Also interdisciplinary healthcare teams have been studied through the lens of complexity science [6]. The relationship patterns between individuals resulting in local interaction strategies that affect the quality of care delivery, the rate of information flow and the adaptability during uncertain conditions have been studied in great detail [8–11]. As such, it has been illustrated that the inter-individual interaction is a driving force and a defining factor for the whole system behaviour. So far, most of the studies have focused on a few - usually three or four - selected attributes of complexity theory, with relationships, self-organization and diversity being the most studied ones [6]. Studies do not, however, systematically evaluate all of the CAS characteristics in a team. Additionally, the learning effect of collaboration, the so-called workplace learning, as an emergent behaviour has been described by focusing on collective competence as a distributed capacity of a system or by describing adaptive practices based upon case descriptions [11–13]. Thanks to the quality of the relationships, feedback loops are created and information is shared which, in turn, influences knowledge growth and generates new behaviour in a team [9]. The factors influencing this workplace learning process require further exploration. With some exceptions, another common feature of most studies is that they report on steady healthcare teams working in one institution, such as hospital or nursing home [6, 11, 14]. An example of distributed teams already described in literature as CAS are the palliative home-care teams (PHCTs) [10, 15]. Internationally, palliative home-care teams have been introduced to deliver expert palliative care to patients at home, in collaboration with the regular primary healthcare professionals [16]. These interdisciplinary teams are performing demanding tasks in ever-changing working environments that require high adaptability of the teams [10, 15]. The network structure, where the collaborating team is geographically spread across different organisations, is distinctive for the PHCTs. Previous research has shown that during this collaboration, workplace learning occurs [15]. Exploring through the CAS lens how this learning occurs as emergent behaviour might provide us with additional insights and pave the way for optimizing the learning network feature of this collaboration.

Therefore this study aims to:

1. Systematically identify all CAS principles as expressed in healthcare providers’ accounts of collaboration in a network structure.
2. Describe the whole-team functioning according to the CAS principles .
3. Explore factors influencing workplace learning in a distributed team as emergent behaviour of a CAS.

## Methods

### Setting and participants

The focus of our study is the healthcare team taking care of the palliative patient at home. We consider the PHCT nurses, the community nurse (CN) and the general practitioner (GP) as the members of this team. The PHCT expert nurses collaborate with GPs and CNs in case complex problems occur involving palliative patients at home. General practitioners carry final responsibility for patient care, PHCT nurses have an advisory role, and CNs are dependent on a GP’s prescription to execute their job. As part of a larger study on collaboration in palliative home care in Belgium [15, 17] we interviewed PHCT nurses, community nurses and general practitioners. In that study, all patients (taken care of by the PHCTs) who died during a three-month period were included as index patients in the study but did not participate themselves. Attending GPs, CNs and PHCT nurses of included patients were invited to participate after the patient’s death.

### Study design and data collection

To study behaviour and interaction, direct observation is the most obvious way of gathering data. However, to understand the ways in which members of healthcare teams operate on a day-to-day basis using the CAS principles as a theoretical framework, insights into the members’ perceptions of their daily interaction with each other should be gained. This is because some of the CAS principles cannot be judged through observation of behaviour but require a reflective stance, and are better studied through understanding the individual’s perception of the interaction. For instance, whether an individual’s behaviour is, following the CAS principle, ‘acting according to internalized basic rules’ cannot be judged through observation but can only be understood by analyzing accounts of the team members’ perception of the basic rules and the way they internalized them. Moreover, the individual’s perception of the other’s action or of the inter-individual interaction will shape the individual’s own behaviour. Therefore, we decided to use individuals’ accounts of their perception of team-interaction through interviews. After obtaining informed consent, semi-structured interviews were held focusing on interprofessional collaboration. The interview guide was designed based upon literature on interprofessional collaboration and comprised the following topics: 1. Experiences during the collaboration; 2. Communication with other professionals; 3. Learning from each other during collaboration; 4. Sharing of tasks and responsibility [15, 17]. The interviews lasted between 45 min and one hour, were audio-recorded and transcribed verbatim.

### Data analysis

Step 1, deductive approach: Two researchers (PP, FM) discussed the CAS principles (see Table 1) and agreed on how to perform the coding using the set of principles as coding framework. After this, one third of the interviews were coded independently by both researchers. The interviews were searched for excerpts where CAS principles could be identified. These excerpts were coded according to the CAS principles. Coding was compared and differences were discussed until agreement on all codes was reached. Next, each researcher coded half of the remaining interviews. This will provide the answer to research question 1.

**Table 1:** Features of Complex Adaptive Systems (CAS) described as team characteristics [1, 3]

1. Team members act autonomously guided by internalized basic rules	
Each team member can act in an autonomous way, guided by basic internalized rules. These rules can be expressed as instincts, constructs	
2. Team members’ interactions are non-linear	
Each team member can act autonomously but the actions have an effect on other team members (and vice versa). This is called the interdependence of the team members. These interactions encompass an exchange of information. An important aspect of the interactions is their non-linearity: small inputs may have large effects and vice versa.	
3. The team has a history and is sensitive to initial conditions	
The non-linear effects observed in a team result from the modifying influence of initial conditions on the interactions between components. As a result of evolution in the system, the ‘initial conditions’ for future interactions will be different. As such, a team has a history and a memory, which means that changed conditions are ‘remembered’ by the system.	
4. Interactions between team members can produce unpredictable behaviour	
As the interactions can cause non-linear effects, it is impossible to always predict the behaviour resulting from the interactions. Secondly, since the internalized rules are not necessarily equal for all components, the influencing factors for a cause-effect mechanism are not always clear.	
5. Interactions between team members can generate new behaviour	
A team can display behaviours that cannot be understood by the characteristics of the individual team members.	
6. A team is an open system and interacts with its environment	
Teams are connected with their environment in different ways. Some of the internalized rules come from the environment; if these rules change, the team changes. As such, the emergent behaviours of teams can be seen as adaptations to the environmental conditions, also called ‘self-organisation’. This self-organisation is informed by feedback loops by which the environment feeds the outcomes of the team’s actions back into the system. Next, depending on the scale we use, the environment may be part of the team or act as environment. As such, the borders of a team are not fixed but can open or close as a response to interactions with the environment. Finally, the environment consists of teams as well and they all influence each other. A team and its environment co-evolve during this interaction.	
7. Attractors shape the team functioning	
The actions and interactions of team members are influenced by a set of basic rules as described earlier. Rules push a team member towards a certain action. As a mirror image, attractors attract team members towards a certain action. The trajectory of a team (i.e. the usual pattern of behaviour) is for a great deal determined by its attractors. The precise behaviour of a team on a precise moment is still unpredictable but the ‘usual’ behaviour will always incline towards the attractors.	

Step 2, inductive approach: For step two, the fragments extracted from the interviews and coded per CAS principle were considered as units of analysis to understand the team functioning according to each principle [18]. Per CAS principle, meaning units were identified by PP, abstracted and labelled with a code. These codes were subsequently sorted into categories. The researchers engaged in a process of reflection and discussion during the analysis and referred back to the original interview transcripts on a regular basis. The underlying patterns within the categories will be presented as aspects of team behaviour in the results section under step 2 [18]. By describing the inductively identified categories in light of the team’s daily practice, we will glean the answers to aims 2 and 3.

We used NVivo 10 for the management and analysis of the transcripts.

### Reliability, rigour and credibility

Trustworthiness of the data was increased through investigator triangulation. At regular intervals, researchers compared and discussed data and checked analysis by referring back to the data. Three researchers are well-versed in qualitative research. Reflexivity was used throughout the analysis.

## Results

### Participants

We interviewed 21 PHCT nurses, 20 community nurses and 18 general practitioners. Participants showed a wide variability in terms of age, gender, working experience and practice situation. Details are shown in Table 2.

**Table 2:** Characteristics of study participants

Discipline (N)	Mean age (range)	Gender (male/female)	Working experience (years)	Practice situation (solo/duo/group)
General practitioner (18)	46 (33–65)	12/6	6–38	9/4/5
PHCT nurse (21)	46 (34–57)	3/18	0,5–15	0/0/21
Community nurse (20)	46 (35–57)	4/16	2–35	4/0/16

### Results aim 1

Every CAS principle could be identified in the interview transcripts of the three professional groups. There are differences, however, in the frequency of identifications, per CAS principle and per professional group. Details are shown in Table 3.

**Table 3:** Number of interviews and excerpts per CAS principle with illustration of an interview fragment

CAS principle	Number of interviews where fragments have been found according to the CAS principle (GP/PHCT/CN)	Number of excerpts according to the CAS principle (GP/PHCT/CN)	Example of interview fragment coded under the CAS principle
1. Team members act autonomously guided by internalized basic rules	48 (16/14/18)	190 (40/62/88)	I think I might have a talk to him about this, because, you know, I really don’t like nurses administering drugs without me knowing it. Or give some extra painkillers just like that. (GP)
2. Team members’ interactions are non-linear	11 (1/6/4)	11 (1/6/4)	He broke it off quite abruptly. Erm, the procedure was what she was, yes, euh, I had the impression that he shot the messenger while he was talking about the procedure. If he doesn’t agree with the procedure, then he can question it, but he doesn’t have to shoot the messenger, I think. So erm it ended up us having to ignore the syringe driver. That he was going to see to it himself] (PHCT nurse, after an altercation between a GP and the PHCT nurse on medication dose)
3. The team has a history and is sensitive to initial conditions	44 (10/17/17)	130 (25/45/60)	That’s right, yes yes. Personality, yes, plays a major role in everything. Yes Yes. I see that, if you look at all of our GPs we work with, and those with whom you work occasionally, or those with whom you work very often, then your communication is also very different. (CN) You will be more assertive in the presence of certain general practitioners. How many times have you worked with them? Erm. What are previous experiences with this general practitioner? If you’ve had a very bad experience, then you will also be much more cautious. Then I think: “Well, the previous experience wasn’t good, I have to make sure that this one goes well” (PHCT nurse)
4. Interactions between team members can produce unpredictable behaviour	21 (2/11/8)	38 (2/22/14)	It depends on your openness as a doctor. If you have a closed mentality, then you will receive suggestions that you don’t really need, whether you like it or not. (GP)
5. Interactions between team members can generate new behaviour	25 (5/13/7)	54 (6/31/17)	I go and ask the members of the palliative team. Is there a solution to this problem? And there’s also a development in this and those people are more aware of it. If we try to do it well, each from our own expertise and our own training background, you will reach a higher level together] (GP)
6. A team is an open system and interacts with its environment	31 (7/12/12)	70 (16/24/30)	But I think it’s actually because of us that they can be admitted (PHCT), that’s not an obvious a step to take. So often, it’s the hospital that takes the first step. The patients are discharged from the hospital and then we have to take care of the aftercare. They are usually aware of the existence of a palliative service, but I still think we take the first step most of the time (CN) And there are those who know it very well, of course. We also have several GPs who have followed the course (on palliative care), who are also well-informed. Sometimes, when I enter the place they approach me and say, ‘Have a look, I’ve done that calculation in such or such a way, what do you think. In consultation, that’s great, isn’t it? But there are different types of general practitioners. Yes, there is still a lot of work to be done] (PHCT)
7. Attractors shape the team functioning	52 (14/18/20)	180 (26/50/104)	In the case of older doctors, it is usually the case that we try to give them the impression that the decision is theirs, but in most cases, we have talked them into it. How can we give them the sense that they made the decision while arriving at a point where it becomes doable for our patient? (CN)

### Results aim 2

Below, we present the emergent patterns within the categories for each of the CAS principles.

#### 1. Team members act autonomously, guided by internalized basic rules

Our study results revealed three basic rules shaping the team members’ professional attitude and the way they engage in the collaboration.

- Participants in our study clearly stated that their mission in healthcare was to focus on the patient and the quality of care. *‘We are here for the patient’* is the most important basic rule for the three professional groups in our study, making it the driving force for team collaboration by sharing complementary expertise. The focus on patient care, along with the willingness to act in the best interests of the patient makes team members acknowledge each other’s expertise and allow them to express their opinions on patient problems within their area of expertise or seek advice with other team members in cases of indecision without interference of professional hierarchy. In cases of team members not sharing their expertise or acting on their own, other team members will restore communication without damaging interprofessional relationships.
- The awareness of *GPs carrying final responsibility* is a second basic rule within the context of the healthcare team in our study. As such, nurses cannot initiate or adapt medication or other therapies without seeking the GP’s consent. The PHCT nurse confirms the GP’s central position by taking the time necessary to deliberate with them the treatment options before meeting the patients and their families.
- A third basic rule in the context of our study is that *tasks and responsibilities need to be clear for everybody*. Having said that, every team member is responsible for their own tasks and duties, although these are not always clearly defined and agreed upon. For instance, monitoring the effect of changes in medication dose on patient’s pain level can be done by all professionals involved. Clear agreements are needed in order for therapy adjustments to be followed up efficiently. Even when tasks and roles are being negotiated and agreed upon within a team according to the patient’s care needs, some external rules are not to be violated, for instance certain protocols on complex procedures, like palliative sedation.

#### 2. Team members’ interactions are non-linear

- *Escalating communication conflicts* were identified as examples of non-linear interactions. One PHCT nurse reported explaining to a GP why they could not assist them in a euthanasia case of a non-terminal patient, because these cases do not belong to the target group of the palliative care team (thereby correctly stating the limits of the team’s official mandate). As a response, the GP reacted in an angry manner and subsequently decided to cease collaboration with the nurse involved.

#### 3. The team has a history and is sensitive to initial conditions

##### History

Several aspects of a team’s history influence the current collaboration:

- *Previous positive experiences* of perceiving the complementarity of each other’s expertise in providing good quality patient care makes professionals trust one another and share tasks and responsibilities more easily. The resulting mutual respect of each other’s knowledge and expertise creates a positive working atmosphere and prevents role conflicts. In case of disagreements on treatment options or differing views on care aims, open and immediate communication is initiated. Positive experiences allow for professionals making a mistake without being blamed by other team members.
- *Previous negative experiences*, however, like nurses acting autonomously without consulting the GP, or GPs neglecting to inform CNs sufficiently on the patient’s medical status or ignoring expert palliative care advice, result in an atmosphere of distrust and lead to professionals acting on their own without sharing tasks. This results in a fragmented care delivery, often confusing both professionals and patients about general care aims. In case of disagreements or differing views, there is insufficient communication and professionals act according to their own views on care without consultation or support for their views from other professionals. As such, a history of poor collaboration makes team members judge each other in a harsher way.
- *Knowing each other* either through previous collaboration or on a personal level facilitates communication and establishes a basic sense of trust in each other’s competences. You get to know the other’s strengths and weaknesses, which results in tailored communication and collaboration.
- *The communication history* also has a major impact. A tradition of systematic and frequent communication facilitates the initiation of a deliberation in case of problems. Previous communication problems, like a GP being repeatedly unavailable for consultation or unwilling to negotiate treatment, cause nurses to find support with other team members, thus excluding the GP from the interaction.

##### Initial conditions

Alongside the sometimes longstanding and continuous history of a team, the initial starting conditions for every new collaborative episode influence the way team members interact with each other.

- *When a GP is reluctant to share the care for, and the information* about, their patient at the start of the collaboration with the PHCT nurses (e.g. when family members ask for the PHCT nurses’ involvement or when hospital services initiate the collaboration when the GP does not feel the need), the interprofessional interaction is less spontaneous throughout the period of collaboration and the PHCT nurses hesitate to make therapy suggestions or discuss care goals. When, however, the GP welcomes the PHCT nurses, or invites them to collaborate, and provides them with insider information about their patient, the PHCT nurses will be more inclined to share all their insights and define shared aims and goals for patient care.
- A team of professionals trusting each other based upon previous collaborations and showing a willingness to collaborate from the start can, therefore, launch a *kick-off meeting* to define care goals and aims before the start of the collaboration. This ultimately leads to more open and constructive communication throughout the collaboration.
- *The composition of the team* at the start of the collaboration influences team members’ interactions and the way the team functions. Community nurses having displayed knowledge and expertise on palliative care in previous collaborations receive the GP’s trust and are invited by them to discuss treatment options. Similarly, GPs (e.g. younger doctors who received palliative care training as part of their undergraduate studies) who have proved knowledgeable before, encourage more open communication and straightforward deliberation with CNs than GPs without expertise. The same open interaction is facilitated by GPs addressing CNs as peers from the start while GPs stressing professional hierarchy at the beginning of the collaboration hinder open communication.

#### 4. Interactions between team members can produce unpredictable behaviour

- *Crossing task boundaries or tightening them* can be the unexpected result of communication on task and role agreements. A GP telling the PHCT nurses that the former will be in charge of medication decisions often receives unsolicited therapy advice from the PHCT nurses who judge the GP’s intentions incorrect, based upon their own expert knowledge. Similarly, PHCT nurses questioning the GP’s decision in a professional way, with good care in mind, can be told not to interfere and not to engage in future therapy discussions.
- *Professionals sometimes ignore their own knowledge and expertise* and act in suboptimal ways without apparent reason. PHCT nurses often accept that GPs ignore their advice, without confronting the GP. They prefer to provide suboptimal care to the patient (according to the GP’s decision) and to closely monitor the patient and report on suboptimal results, eventually leading to therapy adjustment as was their first choice. The reason to act in this manner is not to damage the collaborative relationship with the GP, which might harm future collaborations for future patients. Similarly, CNs often tend to accept GP’s choices that are in contrast with their own views, without commenting upon it. Their reason is that they are dependent on the GP (for prescriptions, for example) for their daily work.

#### 5. Interactions between team members can generate new behaviour

- During interaction and collaboration, professionals learn from each other. This *workplace learning, the acquisition of new skills as an individual or as a team, can lead to a new way of functioning* and is major emergent behaviour resulting from the collaboration. Receiving advice from experts in the team makes team members less dependent in the future and the interaction (advice-seeking behaviour) sometimes diminishes or changes its character. Complex procedures (e.g. palliative sedation or paracentesis) can be executed by a team, even when none of them have ever done it, but combining competences and trust in one another makes the team accomplish the task.
- *New communication strategies* is a second type of emergent team behaviour. Teams with only ad hoc and one-to-one communication may organize whole-team meetings in case of conflicts or as a form of debriefing in the case of complicated collaboration.

#### 6. A team is an open system and interacts with its environment

- *External factors* influence the collaboration. When the GP is less available (e.g. due to workload in general practice in winter time), the PHCT nurse takes over the coordinating role from the GP. As such, the external conditions (e.g. flu epidemic in wintertime causing the GP’s reduced availability) pare down the interaction between GP and PHCT nurses and lead to a reshuffling of tasks and responsibilities in the team. The organisation of out-of-hours service (usual care not available during weekend) triggers initiatives within the team, like GPs sharing their private phone numbers with PHCT nurses, leading to a different way of interacting and communicating. Similarly, anticipating potential problems during the weekend, palliative care teams prepare sets of emergency medication and standing orders.
- *The educational system* influences the collaboration. While better education in palliative care for GPs facilitates discussions with PHCT nurses – indeed, the lack of interprofessional training during undergraduate medical training inhibits effective teamwork - learning to use protocols and guidelines results in less flexibility for the team to decide on how to deliver care. Published guidelines, however, are useful for PHCT nurses to prepare a discussion with GPs.
- *Organizing and financing the healthcare system*. Extra fees for care delivery to palliative patients was mentioned by CNs and GPs as stimuli or compensations for the time-consuming interactions and collaboration. Since community nurses are dependent on GPs for their work (they need prescriptions to be allowed to provide care), they are careful when commenting upon GP’s decisions or actions.
- *Mass media and ideas of the general* public can influence the team dynamic in complex cases like euthanasia (more requests) or medication use (e.g. morphine is lethal), resulting in discussions and more intense team deliberation.

#### 7. Attractors shape the team functioning

Three main themes emerged as attractors that shape the actual day-to-day team operations and care delivery at operational level: quality of patient care, interprofessional relationships and personal and professional wellbeing are goals that team members try to achieve.

- The *quality of patient care delivery* is the main attractor to initiate a collaboration and to shape the collaboration. In order to reach high-quality and comprehensive patient care, professionals combine their complementary knowledge and skills. Acknowledging and respecting each other’s competences often results in deliberation and shared decision-making on treatment plans as peers: professional hierarchy in these cases can be overcome by focusing on expertise instead of on professional background. Some GPs who present themselves as being hierarchically superior in daily practice are willing to make a shift in this behaviour and accept PHCT nurses’ advice in case of complex patient problems. In cases where the attractor of patient care is less present and team members experience rivalry between professions with regard to expertise, collaboration is hindered. Communication as a specific kind of interaction is also influenced by this attractor. Scheduled weekly team meetings are complemented by ad hoc phone calls or supplementary team meetings in case of complex patient problems or when team members do not share the same views on care and care aims.
- *Interprofessional relationships* are a second attractor as they are highly valued between team members. Professionals cover up for each other in case of little mistakes or miscommunication, thereby strengthening the professional relationships. GPs and PHCT nurses sometimes meet before jointly visiting the patient to agree on treatment plans. This is to avoid bedside discussions that might harm the trust of the patient in one or the other and hinder future interprofessional collaboration. In case of conflicting views on treatment options, PHCT nurses often avoid confronting discussions with GPs, and with a view not to endanger the relationship, they prefer to take up the nurses’ role and report on their observations of symptoms in great detail so as to guide GPs to treatment adaptations. Community nurses state doing the same, except when the problem in hand is clearly within the nurses’ expertise e.g. wound care. In those cases, they have no problems contradicting the GP.
- *Personal and professional wellbeing* is a third attractor, shaping the interaction between team members. Professionals mention seeking support with others for a debriefing after an emotional experience (e.g. death of a patient) or after a conflict with the patient or their family. After a collaboration episode (i.e. after the death of a patient), there is often a palliative care team debriefing to evaluate the care delivery. Some GPs regret not being invited to these, because they sometimes feel the need for a concluding talk. Knowing or feeling that they are doing the right thing, and thus avoiding moral distress, brings PHCT nurses to adhere to protocols for complex situations like palliative sedation. They use the protocol in their communication with the GPs to plan task execution. Most professionals prefer not to carry responsibility on their own but to share the burden and seek support or availability of others, even during out-of-hours service. A distinct aspect of professional wellbeing is the CNs’ dependency on GP’s prescriptions, resulting in CNs being careful in addressing GPs and being reluctant to contradict them. This might lead to less professional satisfaction in case of CNs feeling pressurized to perform actions they do not agree with.

### Results aim 3

The interview analysis revealed factors influencing the information exchange and the sharing of expertise within the team. Both are fundamental prerequisites for workplace learning. These factors are described in Table 4.

**Table 4:** Facilitating and hindering factors for information exchange and sharing of expertise, according to the CAS principles

Facilitating factors for information exchange and sharing of expertise (CAS principle)	Hindering factors for information exchange and sharing of expertise (CAS principle)
Sharing the same mission of delivering quality care – the willingness to act in the patient’s best interests stimulates discussions and shared-decision making (1)	
Professional hierarchy – PHCT nurse spends time deliberating treatment options with GP (1) Creating horizontal collaborative relationships from the start facilitates open interaction (3)	Professional hierarchy – nurses acting autonomously without deliberation results in atmosphere of distrust (3) Doctors stressing hierarchy structure might hinder open communication (3). Nurses being dependent on doctors for their daily work and therefore hesitate to comment upon doctor’s decisions even when they disagree (4)
	Unresolved communication conflicts (2)
Previous positive experiences resulting in mutual respect of each other’s knowledge and expertise (3)	Previous negative experiences – GPs insufficiently informing CNs on patient’s medical status or ignoring expert palliative care advice results in atmosphere of distrust (3)
Knowing each other’s strengths and weaknesses results in tailored communication (3) Doctor’s education in palliative care facilitates discussions with PHCT experts (6) Using practice guidelines helps nurses prepare a discussion with doctors (6)	Lack of interprofessional training inhibits effective teamwork (6)
Acknowledging and respecting each other’s competences results in deliberation and shared decision-making as peers (7). Valuing interprofessional relationships trigger anticipatory interprofessional communication in complex cases to avoid bedside discussions (7)	Nurses sometimes avoid confronting doctors with their differing views not to harm relationships. This results in missed learning opportunities (7)
Tradition of systematic and frequent communication facilitates the initiation of a deliberation in case of problems (3)	Communication problems in the past like being unavailable for others or unwilling to negotiate treatment excludes professionals from future interaction (3)
	Unwillingness to collaborate or not feeling the need to collaborate at the start (3)
Sharing information prompts the recipients of information to share information as well (3)	
A kick-off meeting at the start of the collaboration leads to better communication throughout the collaboration (3)	
Extra fee compensates for time-consuming interactions (6). Mass media and general public ideas trigger more frequent and intense team discussions on complex cases (6)	Unavailability due to workload, time restraints diminish interaction (6)
Striving for personal and professional wellbeing triggers interprofessional debriefing after emotional experiences or conflicts with patients (7) Nurses’ hesitation to take up responsibility on their own makes them seek support and deliberate with others, even during out-of-hours service (7)	

## Discussion

Our study described the team members’ interactions based upon the complexity science framework. We explored the origin of healthcare team behaviour and the factors influencing workplace learning as emergent behaviour. Studying healthcare team members’ perception of their interprofessional interaction during day-to-day teamwork through the lens of complexity science helps us to understand how and why healthcare professionals behave in this way as “perceptual information guides our decisions and actions, and shapes our beliefs” [19]. This understanding cannot be derived from studies describing behaviour through observation. Our study allowed us to map internalized views of healthcare providers that define team behaviour. Healthcare teams do not always function as a CAS. In clinical situations where problems and their solutions can be addressed by drawing on procedures and guidelines, teams work in a plan-and-control way, instructions are being given and executed in a straightforward way. Under circumstances where there is uncertainty about how to best deal with the situation, thinking outside the box and trying out different approaches is the most efficient strategy [20]. In these cases, teams work as a CAS. In our study, we found examples of the plan-and-control actions; however, for the purpose of the paper we only focus on accounts of collaborative practice as a CAS [20, 21]. Some of our results are confirmed by literature, both from studies using complexity science as a framework and by studies based upon general learning theories. We will first describe them briefly. Later on, we will focus on the functioning of the team as a learning network and on the understanding of the origin of workplace learning as an emergent behaviour, as we believe that complexity theory can advance our understanding of this theme [5].

Addressing the first study aim, we can state that the CAS principles can be identified in team members’ accounts of their perception of the way they interact on a day-to-day basis in the team. We notice that every CAS principle is to be found in the three professional groups’ accounts of their perception of team interaction, indicating the relevance of the principles for each professional background. Principle number 1 (team members act autonomously, guided by internalized basic rules) and 7 (attractors shape the team functioning) are illustrated by more fragments from more interviews than the other principles. A reason might be that these principles are most relevant for the daily collaborative practice of the team and, as such, most discussed and most accessible for reflection during the interviews. The shared aim and purpose of teamwork is a major topic to be actively discussed repetitively by team leaders, as is the construction of shared mental models in order to collaborate effectively [22, 23]. These two principles have a structuring quality on the team functioning and behaviour. This structuring quality can also be found in principle 3 (A team has a history and is sensitive to initial conditions) and principle 6 (A team is an open system and interacts with its environment), and both have a large number of fragments and interviews. The other, lesser illustrated principles (e.g. 2. Team members’ interactions are non-linear), focus more on the result of the structuring principles and may be less prone to be discussed in daily practice. Equally, team survey instruments do not always capture these dynamic aspects of behavioural processes and emergent states but focus only on the tangible end result [24].

Addressing the second study aim, we can say that healthcare team functioning can indeed be described according to the CAS principles based upon the team members’ perception of their day-to-day interaction. Moreover, this way of analyzing the interviews adds an explanatory understanding of the origin of team functioning based upon individual’s interactions on top of the descriptive representation in literature [25, 26]. Below, we discuss the different principles according to the frequency they have been identified with within the participants’ accounts.

The themes represented under CAS principle 1 (Team members act autonomously guided by internalized basic rules) and 7 (Attractors shape the team functioning) relate to the issue of professional and interprofessional identity. Internalized basic rules and the choice of attractors, to some extent, define people as professionals. During professional identity formation, the characteristics, values and norms of the profession are internalized, which results in an individual acting accordingly [26, 27]. This relates to CAS principle 1. Individuals can, however, develop a dual identity, encompassing both a professional and interprofessional identity [28, 29]. This interprofessional identity builds on the professional identity and helps individuals as they work in teams become part of a collective identity, with agreed goals for the delivery of high-quality patient care [30] . This relates to the attractors of CAS principle 7, which shape the team functioning.

The Interprofessional Education Collaborative (IPEC) has introduced Core Competencies for Interprofessional Collaborative Practice to guide educationalists in designing interprofessional curricula and provides us with an important framework to look at interprofessional collaboration [31]. Sharing one’s personal values with team members and trying to find common ground for a shared aim in teamwork matches competencies 1 (Values/Ethics for Interprofessional Practice) and 4 (Teams and Teamwork) of the Core Competencies for Interprofessional Collaborative Practice of the competency framework. Respect for one another’s values in teamwork is also one of the most commonly assessed dimensions of teamwork survey instruments, as described in a recent review [24]. As such, the patterns found in our results match the literature in identifying major foci for collaborative practice and add an extra layer of meaning to the competencies and measurement instruments described above. The insights from our study can thus be used to clarify and illustrate at a practice-based level the competencies and measurement instruments during interprofessional education or evaluation of team functioning. Understanding how team members’ interaction influences team behaviour is of importance in designing team training and crew resource management training [32, 33].

Another well represented CAS principle in our study is number 3 (The team has a history and is sensitive to initial conditions). The fact that all professional groups mention previous experiences as a major factor shaping current collaboration illustrates the importance of this principle and has been described before [34]. The team culture and the leadership style influence the way experiences contribute to the functioning of a team [35]. In accordance with the IPEC Core Competency 4 (Team and Teamwork), the results of our study call for due attention to team composition and longitudinal collaborative experiences. This is of importance in fixed teams, like those on hospital wards, but equally and more challenging in teams with ever-changing compositions, the so-called fluid teams, as is often the case in primary-care settings, where team composition is decided upon according to the patient’s care needs [21]. Additionally, the initial conditions of a single collaborative episode seem important. Therefore, regular team meetings to discuss the collaboration, and not only the patient care, are of great value. These discussions need to make initial conditions explicit but also serve to regularly evaluate collaboration as building on to the team history and preparing the next initial conditions for a future collaborative episode [22, 35]. Although team design and group cohesion, as part of the team’s history, receive attention during team evaluation, the focus on initial conditions modulating a team’s behaviour could be addressed more explicitly, especially in larger collaborative groups or fluid teams [24]. A major aspect of teamwork, as mentioned by our participants, is the agreement on tasks and responsibilities (see CAS principle number 1 – results step 2), and reflects IPEC Core Competency 2 (Roles and Responsibilities) [31] and one of the most commonly assessed dimensions of team measurement instruments [24]. Even though this aspect should be a primary topic on team meeting agendas to prevent conflicts on this issue, it does not always seem to receive the attention it deserves [25, 36, 37]. Lack of clarity on roles and responsibilities hampers effective collaboration [38]. Our study shows that the discussions and agreements on tasks and responsibilities are linked to one’s internalized basic rules, which may partly explain the sometimes challenging team discussions on this topic.

CAS principle number 6 (A team is an open system and interacts with its environment) stresses the interaction between the team and its environment. Working conditions based upon the organizational culture (e.g. communication strategies) or the broader societal rules (e.g. nurses being dependent on doctors for work prescriptions) are mentioned by participants in our study as moderating the team members’ interaction. Context and team culture are known to influence team functioning [35]. Team managers should be aware of this and take the interaction between their team and the broader context into account when discussing team functioning [39].

CAS principle number 5 (Interactions between team members can generate new behaviour) describes the new behaviour a team can show as a result of the interprofessional interaction (e.g. a whole-team meeting can be scheduled instead of relying on the ad hoc, one-to-one communication a team is used to having after a team conflict due to the fact that information on therapy decisions is not communicated adequately). In our study, we also found aspects of workplace learning, meaning the acquisition of new knowledge and skills during collaboration. A recent literature review on workplace learning in primary healthcare describes learning characteristics matching some of the CAS principles, like the influence of hierarchy and of contextual conditions on workplace learning [40]. Creating the conditions to foster workplace learning can shape the emerging team behaviour to optimize functioning and quality of care delivery. This also relates to principle number 6 (A team is an open system and interacts with its environment) as the working conditions are e.g. dependent on the organizational culture and influenced by the culture of educational institutions.

CAS principles number 2 (Team members’ interactions are non-linear) and 4 (Interactions between team members can produce unpredictable behaviour) are the least present in the participants’ accounts of collaboration. On the one hand, both principles 2 and 4 are described by complexity science as shaping normal CAS behaviour. As such, the general team’s behaviour (outside conflict episodes) might also be based upon these. This could not be illustrated, however, with the results of our study. On the other hand, unpredictability and non-linearity may be associated with team conflicts and moral distress. For instance, in order not to harm interprofessional relationships, CNs often hesitate to confront GPs with differing views on care plans, resulting in the perception of suboptimal care delivery by the CNs, ultimately leading to moral distress and professional dysfunctioning [41]. This relates to the overlap and occasional conflict we noticed between CAS principles 1 and 7. The internalized basic rules, influencing healthcare professionals’ identity as a healthcare provider, guide their actions and make them behave in a certain preferred way. This personal preference can sometimes be in conflict with team attractors requiring different behaviour or working strategies [41, 42]. Professionals can modify their behaviour according to the context and the needs of the situation. When team attractors diverge too much from a professional’s preferred behaviour or personal attractor (intrinsic motivation), tension can arise ultimately, leading to reduced professional well-being or team conflicts [43]. The management of the above-mentioned team conflicts relates to IPEC Core Competency 3, Interprofessional communication, and includes, among others, the subcompetencies of conflict resolution and feedback giving.

When it comes to the third study aim, we found many factors facilitating or hampering the information flow and the sharing of expertise, as fundamental conditions for workplace learning. Many of the factors (e.g. sharing the same values and goals, installing horizontal collaborative relationships) have been described already in literature using complexity theory or other conceptual frameworks [44–48]. Some factors however, such as contextual factors of extra fees or the dependency of nurses on doctors’ prescriptions for their job, are less known for influencing information exchange. These factors seem to be specific of the ever-changing team situation in the context of our study and need further exploration. In a similar way, framing the notion of personal wellbeing, acting as a trigger for a debriefing session after emotional or conflict experiences or as a condition for fostering workplace learning, requires further exploration. Finally, some participants stated that good interprofessional relationships, usually seen as the backbone of open communication, resulted at times in hampered communication [49]. While a good and trusting relationship is usually mentioned as being a prerequisite for open and effective communication, our results show that prioritizing this relationship can be done to such an extent that it prevents open communication [50]. This occurred, for instance, when nurses did not want to open a discussion on a doctor’s treatment decision in order not to jeopardize their good relationship with them, even when they were convinced that their decision was not correct. The ways in which health caregivers strike a balance between quality of patient care and safeguarding a good interprofessional relationship as attractors for professional behaviour requires further exploration.

A limitation of the study is that we only have data from one specific context. The comparison with literature, however, shows that our results might be generic and transferability to other contexts might be feasible, although this should be done with the necessary caution. Another limitation might be the fact that we performed a secondary analysis of interviews, conducted within another study. However, the focus of the primary study was similar to the current one, namely interprofessional collaboration. Moreover, we reached data saturation for most of the CAS principles (with ‘non-linearity’ as an exception), which illustrates the wealth of data.

A strength of our study is that we provide an explanatory model of team functioning based upon complexity science while looking at the perceived interaction of team members. Credibility and trustworthiness of the results are guaranteed by the strict analytical procedure on data from three different professions with a wide variety of personal characteristics, and executed by an interdisciplinary team [18, 51].

### Implications for practice and research

Some implications for education and practice can be gleaned. First of all, our results can provide educators with an extra dimension of the IPEC Core Competencies. This proves that these should not only be acquired at an individual level but be explained and trained, taking the interactional origin of the competence-related behaviour into account. As such, team training, with due attention for the perception of interaction, might be of value. Looking at team functioning through the lens of complexity theory emphasizes the value of team training, next to that of individual professional training, as has been generally acknowledged in the literature and has been operationalized in training models such as the TeamSTEPPS [52]. Secondly, team leaders and team managers might try to frame team drivers, shared focus and aims within the CAS principles. For instance, making professional behaviour explicit as being the result of internalized basic rules or attractors might facilitate team communication and conflict management. Additionally, our study illustrates how team attractors can modulate behaviour and therefore attractors (existing and new ones) are worth exploring and identifying during team training. While trying to induce change at a systems’ level, often emphasis is being placed on overcoming barriers. Complexity theory suggests, as is evident from our data and other studies, that focusing on endorsing existent or installing new attractors might be more efficient [53]. A review of workplace learning during collaborative practice in primary care identified possible attractors (e.g. the willingness to learn from each other triggers open communication and respect for the other’s views) that might be used as a source of inspiration in team training [40]. Thirdly, as workplace learning during practice is a substantial part of continuing professional development, creating the conditions to facilitate learning as emergent new behaviour requires attention from team leaders and managers.

Future research needs to confirm these results in other contexts. Also, the overlap and potential conflicts we noticed between CAS principles 1 and 7, where team attractors sometimes overrule individual internalized basic rules, should be further investigated. The motivation to do so needs to be investigated, as well as the effects of these conflicts on the professional well- being of healthcare providers as overruling aspects of one’s professional identity might lead to moral distress and professional dysfunction.

## Conclusion

This study provides us with insights into the origin of team functioning by explaining how patterns of interactions between team members define team behaviour. Viewing healthcare teams as complex adaptive systems may offer explanations of different aspects of team behaviour with implications for education, practice and research.

## Abbreviations

CAS: Complex adaptive system
CN: Community nurse
GP: General practitioner
IPEC: Interprofessional education collaborative
PHCT: Palliative Home Care Team
